# Understanding urbanization: A study of census and satellite-derived urban classes in the United States, 1990-2010

**DOI:** 10.1371/journal.pone.0208487

**Published:** 2018-12-26

**Authors:** Deborah Balk, Stefan Leyk, Bryan Jones, Mark R. Montgomery, Anastasia Clark

**Affiliations:** 1 CUNY Institute for Demographic Research, City University of New York, New York, New York, United States of America; 2 Marxe School of Public and International Affairs, Baruch College, City University of New York, New York, New York, United States of America; 3 University of Colorado, Boulder, Colorado, United States of America; 4 Population Council, New York and Stony Brook University, Stony Brook, New York, United States of America; Tel Aviv University, ISRAEL

## Abstract

Most of future population growth will take place in the world’s cities and towns. Yet, there is no well-established, consistent way to measure either urban land or people. Even census-based urban concepts and measures undergo frequent revision, impeding rigorous comparisons over time and place. This study presents a new spatial approach to derive consistent urban proxies for the US. It compares census-designated urban blocks with proxies for land-based classifications of built-up areas derived from time-series of the Global Human Settlement Layer (GHSL) for 1990–2010. This comparison provides a new way to understand urban structure and its changes: Most land that is more than 50% built-up, and people living on such land, are officially classified as urban. However, 30% of the census-designated urban population and land is located in less built-up areas that can be characterized as mainly suburban and peri-urban in nature. Such insights are important starting points for a new urban research program: creating globally and temporally consistent proxies to guide modelling of urban change.

## Introduction

In 2014, urban dwellers were estimated to account for 54 percent of the world's total population, with that percentage projected to grow to 66 percent by 2050 [1]. Yet the meaning of urban in such often-cited figures is decidedly unclear: there exists substantial variation across countries in the urban definitions adopted by their statistical authorities, and countries commonly change definitions over time [2]. Even in the United States, where urban definitions have been well documented and accompanied by census data available in fine spatial detail, the evolution of concepts and measures over the past few decades has made it difficult to craft a consistent analytic account of urbanization [3,4]. In other countries lacking comparable documentation and data, and certainly when comparisons are made across countries, the barriers to understanding urban change can be formidable (see, for example, continental [5] and global [6] efforts to harmonize urban delineations).

In the demographic research community, urban population has been mainly defined in terms of population density, contiguity and size, with urban measures often enriched by consideration of socioeconomic indicators of connectivity such as commuting zones [7,8,9,10,11]. Substantial differences exist across countries in the criteria applied to define populations and land areas as urban [1]. According to the UN [1], 20 percent of countries use a purely population-count based definition, while about 28 percent of all countries have adopted a single administrative criterion. Others employ functional definitions associated either with the presence of urban or the absence of rural agricultural characteristics; and many draw upon a combination of criteria (e.g., China, USA, India, Philippines, Sweden)–which may include housing density, land use types and other factors in addition to population size, density, and administrative or economic status. The density or count threshold used to identify urban populations in any given country often appears to reflect the country's overall population size and average density. For example, any community of more than 2,500 people is considered urban in Mexico, whereas in Nigeria, a settlement defined as urban must exceed a population of 20,000. Density-based definitions are often supplemented with contiguity indicators, allowing for the inclusion of low-density communities when they border larger and denser settlements. In this paper, as in the US Census, we refer to ‘traditional’ population density measured as population/area, sometimes referred to as net density. Alternative measures of density such as urban or residential densities, particularly for cities, are described elsewhere [12]. Additionally, some measures of urban area give explicit consideration to governmental jurisdictions and boundaries, as in the case of metropolitan statistical areas in the US which are formed from contiguous sets of counties.

In contrast, urban land has been defined through land-use and land-cover measures in keeping with the methods and concepts of this research community [13,14,15,16]. Numerous studies suggest that no more than 3% of global land is urban [17,18,19], and that in the near term, urban land is expected to grow twice as fast as urban population [20]. Yet, based on their key argument that not all urban land is built-up, Liu and colleagues [17] find that only 0.65% and 0.45% of the global land is built-up area, and impervious surface, respectively.

Both analytic perspectives have emphasized the gap between the richness of their urban concepts and the limited abilities of the available data to measure these concepts [21,22]. Most low-income countries do not yet produce the spatially-detailed demographic data they would need to adequately differentiate urban and rural populations and monitor change. Data constraints are receding more rapidly in land classification research, which is benefitting from the increasing availability of fine-resolution, remotely-sensed data with global coverage and spanning several decades, enabling scientists to take advantage of advances in classification and analytical tools for change detection. Land can be designated as urban on the basis of different types of low-to-high-intensity development at varying population densities or even (as in the 2010 US Census definition) no population at all. There may exist good reasons for measures of urban people and urban land to differ in coverage [23], but to date these differences have not attracted systematic research attention.

This study is informed by more than a decade of innovative, interdisciplinary work that has combined remote-sensing data with socio-demographic or ecological information to detect and map urban areas and change [19,20,23,24,25,26,27]. In these studies, the remote-sensing derived data–whether of the land use/land cover type or night-time lights [28]–have provided proxies for urban spatial features or boundaries. These studies have not directly compared satellite with census data, in part because they lacked the spatially detailed census data available to us here. Using the US as a test case, we explore the potential of remotely-sensed measures of built-up land to serve as proxies for official census measures of urbanization.

Of course, no remotely-sensed measures can fully substitute for socioeconomically-informed classifications of richly detailed census data, but even in the US such remotely-sensed measures can provide helpful complementary information. More importantly, they have the potential to add significant value in countries that are data-poor or where the ability of current urban-rural definitions to adequately classify settlement patterns is in doubt. Our analysis provides a systematic means of distinguishing peri-urban areas from other types of urban development, which may be useful in future research on finer scale urban development as well as in forecasts of urban expansion. By identifying strengths and weaknesses in the GHSL-derived proxies in a well-documented context, our study contributes to ongoing efforts to develop unified statistics-based definitions of urban from globally consistent time-series [29,30] that could be applied widely throughout the social and natural sciences.

This study has two main goals. First, we assess the agreement between GHSL-based measures of built-up land and census-based urban classifications, aiming to establish whether the former (GHSL) can be used as a suitable proxy for the latter (census data) where the latter are absent. Second, using this unique combination of data layers, we also aim to learn about U.S.-specific trends and processes that influence urban form (e.g., density, sub- and peri-urbanization). We draw out the implications of our finding for using GHSL to fill in for census data (for example, in inter-censal periods) in other settings, including in many data-poor countries and discuss the potential for using such data and methods in spatial forecasts of urban settings. These goals are further articulated in the sections below.

## Approach, materials & methods

In order to explore what satellite-based proxy measures can contribute to defining urban places in a consistent manner, with possible application to data-poor countries, we use the high-quality demographic data of the US Census Bureau to (1) evaluate the ability of one newly available remotely-sensed settlement data product, the Global Human Settlement Layer (GHSL [31]), to approximate census-based measures of urban land and people, and (2) learn about the urban classification system that results from a spatial integration of the two data sources. GHSL classifies built-up land layers using Landsat imagery covering the period from 1975 to 2014. Otherwise similar global data products suffer from limitations either in terms of coarse spatial resolution (e.g., Global Rural Urban Mapping Project; GRUMP [32]), or limited temporal coverage (e.g., GlobCover [33]), Global Urban Footprint [34], GHSL Sentinel [35]). The GHSL dataset, therefore, has the potential not only for understanding and modelling urban patterns and change in ways that other data sets cannot, but may also offer new opportunities for refining population projections and research in field such as disaster management and risk assessment [36]. In the remainder of this section, we describe these underlying data in greater detail, as well as the conceptual framework and data integration methods used to generate our results.

Table 1 provides an overview of the data sources used in this study: census data at the block level for three different census years, 1990, 2000 and 2010 and the aggregate version of GHSL for similar points in time (1990, 2000 and 2014). The table summarizes our integrative effort to identify both “Urban People” (in keeping with demographic perspectives) and “Urban Land” (land-science perspectives): The GHSL data indicate the presence or absence of built structures at fine spatial granularity and are aggregated to express the density of structures (i.e., built-up area proportions) at moderate spatial resolution; the census classification of urban areas is based on population criteria at the census block level that varies in spatial resolution. Whereas the GHSL measures of built-up land are relatively consistent over time, the census-based urban definitions changed considerably over the period we study, underscoring one of the fundamental problems in deriving reliable estimates of urban change. Each data set is described further below. All data used herein are publicly available.

**Table 1:** Input data by type, source, and temporal and spatial resolution.

Urban Feature	Spatial Product	Years	Urban Proxy Definition	Resolution
**Census geography-*Urban People***	US Census blocks	1990	Population density and count dependent.	Variable (delineated by man-made and physical features of the landscape)
		2000	Population count, density and proximity dependent.	
		2010	Population count, density, proximity, and urban land-use dependent.	
**Satellite-based urban proxies-*Urban Land***	Global Human Settlement Layer	c. 1975, 1990, 2000, 2014	Measure of built-up land cover. Urban extents are constructed based on grid cell values meeting a 25%, 40%, or 50% built-up threshold.	304 meters

### What is the census meaning of urban?

At the core of this analysis is the finest-grained spatial unit of analysis for the United States census—the census block, for which there is complete geographic coverage over the last three decennial censuses (1990, 2000, and 2010). Census blocks are delineated by both man-made and physical characteristics of the landscape, such as roads and rivers; in densely populated urban areas they typically comprise actual city blocks. Unlike larger census units such as tracts, blocks can vary widely in total population, which ranges from zero in many instances to several hundred or even thousands of persons in the case of densely populated city blocks. In response to changes in development and population density, the total number of blocks has risen substantially over the past three censuses, increasing from just over 7 million blocks in 1990 to over 11 million blocks in the 2010 census.

At each census, these blocks and the population contained within them are defined as urban or rural according to criteria that have changed over time (see Table 1). The dominant methodological trend over the three censuses has been the gradual elimination of municipal and place boundaries from the Census Bureau’s statistical definitions [3]. At the time of the 1990 census, urban/rural status was based on both total population and density criteria. Cities (which refer to census incorporated or designated places) of greater than 50,000 persons (urbanized areas, or UAs), and all blocks contained in them, were classified as urban. Additionally, any census blocks adjacent to such qualifying territory with a population density over 1,000/mi^2^ were included in the larger urbanized area. Outside urbanized areas, blocks were defined as urban if they were part of census designated places with a population greater than 2,500. For the 2000 census, the use of census incorporated/designated places (cities) as a starting point for constructing UAs was dropped and census blocks became the primary building blocks. By definition, to qualify as a UA a contiguous set of blocks demonstrating population density > 1,000/mi^2^ needed a total population of > 50,000. The count-oriented definition for smaller (<50,000) urban places was also dropped in favor of a new measure known as urban clusters (UCs) which were defined using the same population density criteria as UAs (>1,000/mi^2^). Urban clusters were defined as a core set of contiguous census blocks with a density greater than 1,000/mi^2^ and a total population of 2,500–49,999. Any blocks within UAs and UCs were thus defined as urban, as were any census blocks adjacent (within 2.5 miles) to UAs and UCs provided that their population density exceeded 500/mi^2^. For 2010, the 2000 urban classification scheme was further amended to include some categories of land in industrial and commercial use: non-residential blocks mainly covered by impervious surfaces (pavement, parking lots, and airports) in close proximity (within 0.25 miles) to populated urban blocks within UAs and UCs. In an innovative combination of the demographic and land cover research perspectives, the Census Bureau drew its impervious surface measures from Landsat imagery prepared by the National Land Cover Database [3]. Additional intricacies of the census data definitions can be found on-line.

### What is the meaning of GHSL-derived built-up land?

The Global Human Settlement Layer (GHSL), produced by the Joint Research Center (JRC) of the European Commission, represents a new generation of global built-up land data products, encompassing 40 years of historic change (1975, 1990, 2000, and 2014) at fine spatial resolution (approximately 38 meters, aggregated to 304 meters). More than 40,000 Landsat scenes have been processed in a consistent manner across countries and over time, drawing upon state-of-the-art built-up land extraction methods using advanced machine learning algorithms [35,37]. In their original resolution, the data are binary, indicating either the presence or absence of a built structure in each 38m grid cell [31,38]. The 304m data are constructed from the 38m cells; they record the percentage of the 304m cell that is built-up. (A revised release of these data has been issued at a resolution of 250m, but as these have been resampled from 304m version, we use the 304m data here to avoid any bias incurred during the resampling procedure.) A recent validation study has generally confirmed the accuracy of the GHSL algorithms except perhaps in very sparsely-settled rural regions; for details, see Leyk et al. [39].

### Method: Conceptual framework and data integration

The analytical challenge we face here is how to derive such GHSL-based proxies and critically assess their relationship to census-based depictions of urban land and urban population. Focusing on the US census years of 1990, 2000, and 2010, the aim of our study is to assess how alternative GHSL-based measures of built-up land relate to and agree with census-based classifications of urban areas, concentrating on the spatially most detailed census-block level.

**Fig 1:**
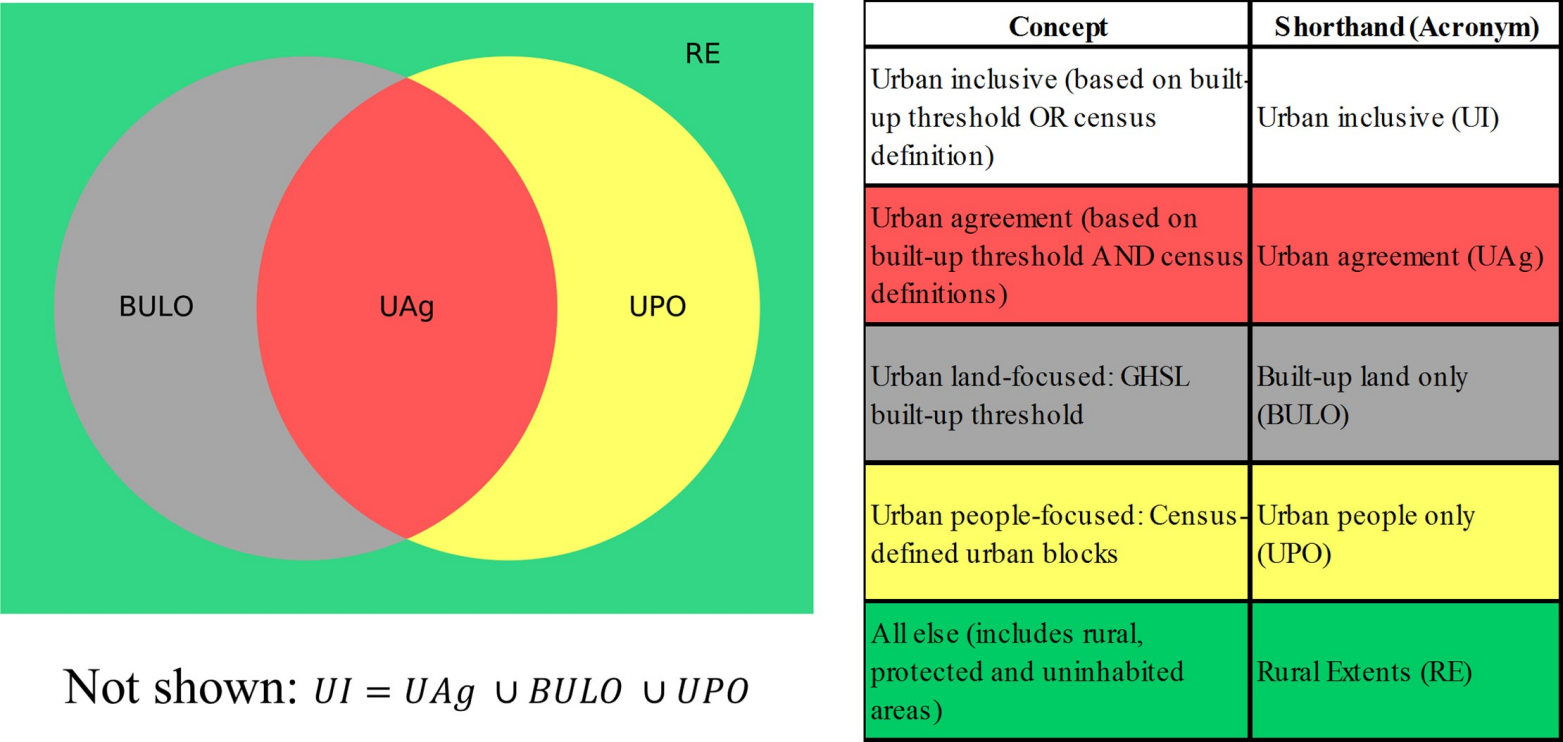
Venn diagram showing possible subsets created from combining layers and resulting classification schema.

The conceptual framework guiding this research is shown in the Venn diagram below (Fig 1), combining census measures of urban population with those of GHSL indicating built-up land exceeding a given density threshold. This spatial overlay of a binary land-cover measure (GHSL) atop a binary census-block classification identifies four classes of land: officially urban and built-up, which we term a class of “urban agreement” (*UAg*); officially urban but not sufficiently built-up (i.e., not meeting the given GHSL threshold), designated as “urban people only” (*UPO*); sufficiently built-up but not officially urban, entitled “built-up land only” (*BULO*); and lastly, residual land that is neither officially urban nor sufficiently built-up, which we describe somewhat informally as “rural extents” (*RE*). This residual class is comprised of the portions of official rural-designated census blocks that also fall below the GHSL built-up threshold. Maps showing these different layers are presented in S1 Fig, and the steps executed to produce them are illustrated in S2 Fig. The combination of the two types of spatial data, and the areas of agreement and disagreement between them, generate an instructively detailed and differentiated picture of urban environments. The three mutually-exclusive classes *UAg*, *UPO*, and *BULO* together make up what we term an “urban inclusive” (*UI*) layer. This summary class extends the official urban totals to include any people and land in the *BULO* zone, that is, built-up area that is not officially urban but which exceeds the given built-up threshold.

Census blocks, each classified as urban or rural by the Census Bureau, provide complete coverage of all national territorial units. Restricting our analysis to the continental, lower 48 states, we overlay the GHSL built-up raster data (304m grid cells) on these census blocks (as detailed in the S1 Fig and S1 Text). The Census Bureau designation of urban is binary—either wholly urban or wholly rural—at the census-block level. In a similar fashion, we adopt a binary coding for the density of structures in each raster cell that intersects a census block, which is coded in relation to a specified built-up percentage threshold. We consider three such thresholds in this analysis– 25, 40, and 50 percent built-up. The most commonly-cited threshold for defining urban land from GHSL is 50 percent [30], but we also use more inclusive thresholds to assess the sensitivity of our results to threshold levels. The EC-standard measure is to date the only formalized threshold in use and therefore we adopt this value as the starting point in our analysis.

Having classified land as conceptualized in Fig 1 and shown in S1 Fig, we proceed to calculate the population living in each of the four classes. One difficulty is that where units of land are concerned, the high resolution of the GHSL often identifies variation in the density of structures within a census block, especially outside city cores where there can be multiple GHSL cells per block. In order to link classes of land to classes of people, we have assumed that the population of the block is uniformly distributed across its geographic area, even in cases where GHSL shows that only a portion of that block is built-up. Specifically, we overlay the four-class spatial distribution described above with the block-based census population data and extract population totals for each layer within each block using a proportional allocation algorithm (by land area, hence applying areal weighting techniques [40,41]). S2 Fig outlines the data-processing algorithm in more detail.

Comparisons of officially-designated urban land and population with the built-up proxy measures are undertaken for 1990, 2000, and 2010. Our analysis is applied to all census blocks in the continental US as a first analytical step to shed light on urban structure and composition at the finest level of spatial granularity, and over time. The results will guide subsequent experiments in future research (currently underway, and presented elsewhere) in which we undertake an analysis of blocks within US Metropolitan Statistical Areas, often used as proxies for cities, and apply these methods to other countries to characterize spatial and temporal variability in the evolution of urban systems.

## Results

We organize our results in four parts. First, we describe our estimates of population and land area for each of the classes we’ve constructed–urban agreement (*UAg*), built-up land only (*BULO*), urban people only (*UPO*), and the residual rural extents (*RE*)–for each of the three census years, using different GHSL built-up percentage thresholds. Second, we elaborate on how built-up area and officially urban estimates compare in order to evaluate how well one type of data can serve as a proxy for the other. Third, derived from this we describe the built-up levels of our different urban classes in order to better understand urban form as well as urban measurement. Fourth, we focus on the transitions in classes over the three censuses to explore the potential for forecasting urban change.

### What do urban classes tell us about measures of urbanization?

In 1990, the population of the continental United States was 247 million; by 2010 the total had risen to 307 million. Official statistics put the urban population of 2010 at 80.7 percent of the total population, up from 75.1 percent in 1990 [42]. For land area (using the Census definition), the urban percentages run from 2.9 percent (in 1990) to 3.6 percent (in 2010) of all continental land (see Table 2). To what extent does adoption of an urban-inclusive (*UI*) perspective alter such percentages? Any additions to the official urban totals would come from the inclusion of what we have termed built-up land only (*BULO*), but as we find, neither the population nor the land area of this class is very substantial. The population inhabiting such land amounted to only 1.2 percent of the continental US population in 1990 (1.5 percent of the urban-inclusive population), declining further to 0.5 percent in 2010 (0.6 percent of the urban-inclusive population). This suggests that the officially designated urban areas include most of the population residing on built-up land portions. (Results for GHSL thresholds of 25 percent and 40 percent built-up are presented in S1 and S2 Tables.) The urban inclusive (*UI*) land area by our estimate has increased from just over 3 percent in 1990 to 3.7 percent in 2010, neither figure being significantly higher than what one would obtain from official urban-designated totals.

**Table 2:** Total population and area, and population density, by urban classification 1990–2010.

		1990		2000		2010	
		Count	%	Count	%	Count	%
**Population (000s)**	**Urban Inclusive (*UI*)**	**188,290**	**76.3%**	**222,504**	**79.6%**	**249,124**	**81.2%**
	Urban Agreement (*UAg*)	131,208	69.7%	152,068	68.3%	170,042	68.3%
	Urban People Only (*UPO*)	54,183	28.8%	68,683	30.9%	77,474	31.1%
	Built-up Land Only (*BULO*)	2,898	1.5%	1,753	0.8%	1,608	0.6%
	**Rural Extents (*RE*)**	**58,447**	**23.7%**	**57,079**	**20.4%**	**57,551**	**18.8%**
**Area (km**^**2**^**)**	**Urban Inclusive (*UI*)**	**235,802**	**3.0%**	**253,925**	**3.3%**	**290,096**	**3.7%**
	Urban Agreement (*UAg*)	78,216	33.2%	89,508	35.2%	108,888	37.5%
	Urban People Only (*UPO*)	146,869	62.3%	150,677	59.3%	170,343	58.7%
	Built-up Land Only (*BULO*)	10,717	4.5%	13,740	5.4%	10,865	3.7%
	**Rural Extents (*RE*)**	**7,574,487**	**97.0%**	**7,556,373**	**96.7%**	**7,520,203**	**96.3%**
**Population Density (persons/km**^**2**^**)**	**Urban Inclusive (*UI*)**	**798.5**		**876.3**		**858.8**	
	Urban Agreement (*UAg*)	1,677.5		1,698.9		1,561.6	
	Urban People Only (*UPO*)	368.9		455.8		454.8	
	Built-up Land Only (*BULO*)	270.4		127.6		148.0	
	**Rural Extents (*RE*)**	**7.7**		**7.6**		**7.7**	

NB: Digitizing differences in the block-level boundaries result in minor (>10 km^2^) in total land area. Built-up area threshold of 50% used. As this analysis is of all urban blocks, no MSA geography is used. See S1 and S2 Tables for other thresholds.

Just over two-thirds of the urban-inclusive population is found in the built-up areas (using the 50 percent built-up criterion) that are officially designated as urban (i.e., the urban agreement class, *UAg*). These are areas that might conventionally be regarded as “core urban” [43], and is where population densities are highest, at 1,561 persons per square kilometer or more in all three census years. However, the *UAg* class accounts for a much smaller share of urban-inclusive land than it does for people, accounting for only about one-third of the *UI* land area in 1990 and 38 percent in 2010. The areas classified as urban people only (*UPO*) contain somewhat less than one-third of the urban inclusive population, exhibiting population densities from nearly 370–456 persons per km^2^ over the period, well below the densities of *UAg* areas. Such officially urban but lower density land accounts for well over half of all urban land area. *UPO* areas are typically found on the outskirts of urban areas, as would be consistent with suburban and peri-urban settlement (see S3 Fig). From 1990 to 2010, this segment of the urban population grew the most in relative terms (by 7 percent in relation to the 1990 benchmark). While core urban and peri-urban are terms that are widely used to characterize aspects of urban form, our classes help to map, contextualize and quantify these aspects in a systematic way. As also shown in Table 2, population densities in non-urban built-up land (*BULO*) are low—not as low as rural densities, to be sure—but well below the population densities of the other two classes of urban land.

Importantly, these characterizations are dependent on which built-up threshold one applies. We have taken 50 percent built-up to be the default threshold, in part because it is the one being used elsewhere [30] as an urban proxy. S1 and S2 Tables present estimates for two more inclusive GHSL thresholds. If the 25 percent built-up criterion were to be adopted, there would be little change in the total urban-inclusive population, but a significant redistribution of this population among the urban classes would take place. A greater share of the urban-inclusive population would belong to the *UAg* category (built-up and officially urban)—81 percent in 2010 as compared to 68 percent in Table 2—and correspondingly the population found in the officially urban but not built-up (*UPO*) class would be 13 percentage points smaller (slightly more than half its size as estimated in Table 2). The population densities of all urban classes would decline with the lower threshold, as would be expected given the correlation between the density of structures and the density of population. The choice of threshold also affects the estimates of land area in each class, but even at the 25 percent threshold, less than 4 percent of total land area would fall in the urban-inclusive category.

### How do built-up and officially urban compare?

Table 3 summarizes the performance of our density-of-structures proxy for officially-designated urban population and land area. Here we compare the more restrictive GHSL threshold of 50 percent built-up to a more forgiving 25 percent threshold. In areas that are built-up by these criteria, the official urban percentage of the population exceeds 98 percent (at the more restrictive threshold) and 85 percent (at the more inclusive one). Similarly, between 87–91 percent of built-up land (50 percent threshold) is officially classified as urban (with about 10 percent less at the more inclusive threshold). In summary, almost all residents of built-up land are officially urban, and a very high percentage of all built-up land is also officially urban. For both population and land, being built-up very nearly implies being officially urban.

**Table 3:** Summary statistics comparing official (USCB-designated) urban areas with authors' GHSL-based urban classification.

		50% GHSL Threshold			25% GHSL Threshold		
		1990	2000	2010	1990	2000	2010
**Of official urban areas (*UAg* + *UPO*)**							
** **	% of the population living in built-up areas (*UAg*)	70.8	68.9	68.7	83.6	82.2	82.3
** **	% of the area that is built-up (*UAg*)	34.7	37.3	39.0	49.9	54.1	56.8
**Of areas that are built-up (*UAg* + *BULO*)**							
** **	% of the population that is officially urban (*UAg*)	97.8	98.9	99.1	86.3	85.1	85.2
** **	% of the land that is officially urban (*UAg*)	87.9	86.7	90.9	76.8	76.4	81.1

But does officially urban imply being built-up? Here, the performance of the GHSL proxy is less impressive but nevertheless instructive. Of all areas officially designated as urban, between 69 to 84 percent of the population (depending on the GHSL threshold) lives on built-up land. Specifically, at the 50 percent threshold, GHSL misses about 30 percent of the official urban population; with the more inclusive threshold this number reduces to about 16 percent of the official urban population, Similarly, of the total land area of official urban census blocks, only 35 to 39 percent is built-up at the 50 percent threshold, by between 50 to 57 percent is built-up at the more inclusive threshold. Being officially urban clearly does not imply being built-up. The two measures are simply not equivalent.

To put this finding in its proper context, it should be recalled that the official urban designations are framed with two population density thresholds, at least one distance threshold (lower densities being allowed for areas contiguous to higher-density areas), and further include consideration of total population size. They can be regarded as a rich, multi-parameter statistical specification of urban-ness. In contrast, the built-up urban proxy that we have constructed here is simple: it has only a single threshold parameter. Therefore, it is not entirely surprising that this proxy fails to capture all the features of complex urban landscapes. Having established the potential of the built-up proxy measures, we see opportunities to improve their performance by devising more flexible parametric specifications, which might mimic the Census Bureau's treatment of population, contiguity, and density, and also to revise estimates of potentially non-urban land and people within officially urban block units. We revisit this point in **Discussion** to draw out the implications for future research. To further assist with that objective, we next examine variation in built-up levels across the different urban classes.

### How built-up are urban classes?

As shown in Table 4 we examine the mean GHSL built-up percentages by class and threshold to better understand the built-up levels of different types of urban environments. By definition, the means for the urban agreement (*UAg*) and built-up land only (*BULO*) classes must exceed the chosen threshold; what is of interest here is the degree to which they do so. For the built-up thresholds of 25, 40, and 50 percent, the *UAg* means of built-up percentages are respectively 66, 75, and 80 percent. Likewise, the urban-people-only (*UPO*) class must by definition exhibit built-up percentages that fall short of the GHSL threshold: the means are in fact far below those thresholds, at 5.5–7 percent, 10–12 percent, and 13–16 percent, respectively. This suggests that a much lower built-up threshold could be applied, either by itself or in combination with rules of proximity (to *UAg* areas), to detect suburban and peri-urban areas within the US. As for the rural residual (*RE*) class, its average GHSL values are below 1 percent irrespective of the threshold, although as mentioned earlier there is reason to believe that these rural results may be based on underestimated built-up percentages in rural settings.

**Table 4:** GHSL % built-up by urban classification.

		Mean Built-up %		
Threshold	Urban Classification	1990	2000	2010
**25**	**Urban Inclusive (*UI*)**	37.4	40.0	41.0
	Urban Agreement (*UAg*)	66.9	66.2	66.1
	Built-up Land Only (*BULO*)	44.7	46.2	43.1
	Urban People Only (*UPO*)	5.5	6.7	7.1
** **	**Rural Extent (RE)**	0.4	0.3	0.4
**40**	**Urban Inclusive (*UI*)**	37.8	40.7	41.7
	Urban Agreement (*UAg*)	75.3	74.8	74.6
	Built-up Land Only (*BULO*)	59.2	60.9	57.9
	Urban People Only (*UPO*)	9.8	11.5	12.2
** **	**Rural Extent (*RE*)**	0.5	0.4	0.4
**50**	**Urban Inclusive (*UI*)**	37.6	40.6	41.7
	Urban Agreement (*UAg*)	80.2	79.7	79.6
	Built-up Land Only (*BULO*)	67.3	69.0	66.4
	Urban People Only (*UPO*)	12.8	14.7	15.6
** **	**Rural Extent (*RE*)**	0.5	0.4	0.4

As Table 4 shows, the average GHSL value for the urban inclusive (*UI*) class exhibits a modest increase over time for each GHSL threshold. Yet, when examined by classes within urban-inclusive, it can be seen that *UPO* is the only class in which the mean built-up percentages are increasing. There are several possible explanations for this: 1) areas that transition into the *UAg* and *BULO* classes are likely to be places that have a lower percent of built-up land (and closer to the thresholds used to construct these classes) than the areas that were previously classified as *UAg* and *BULO*, thus causing the average built-up values to decrease. 2) In *UPO* areas, while some increase in built-up-ness is expected, their average values remain below the respective thresholds consistent with suburban and peri-urban development. 3) Block boundaries change substantially over time as a function of the census-based definition of urban. These changes affect which blocks, or parts of blocks, are included in any given class, contributing to the decreases or increases of average GHSL class values.

### Do patterns of change describe urban transitions?

We now examine the changes in the urban status taking place over the course of one to two decades. The levels of built-up density (Table 4) and the sheer durability of structures, make it unlikely that built-up areas will lose enough structures to fall below a given threshold in the space of a few decades. Transitions from below to above the density thresholds are more likely, at least in cases of rapid development of sparsely settled land. The main changes of interest, therefore, are in those areas that involve the sparsely built-up but urban places in the *UPO* category and the built-up but non-urban places (*BULO*) whose status might be revised by the Census Bureau.

**Fig 2:**
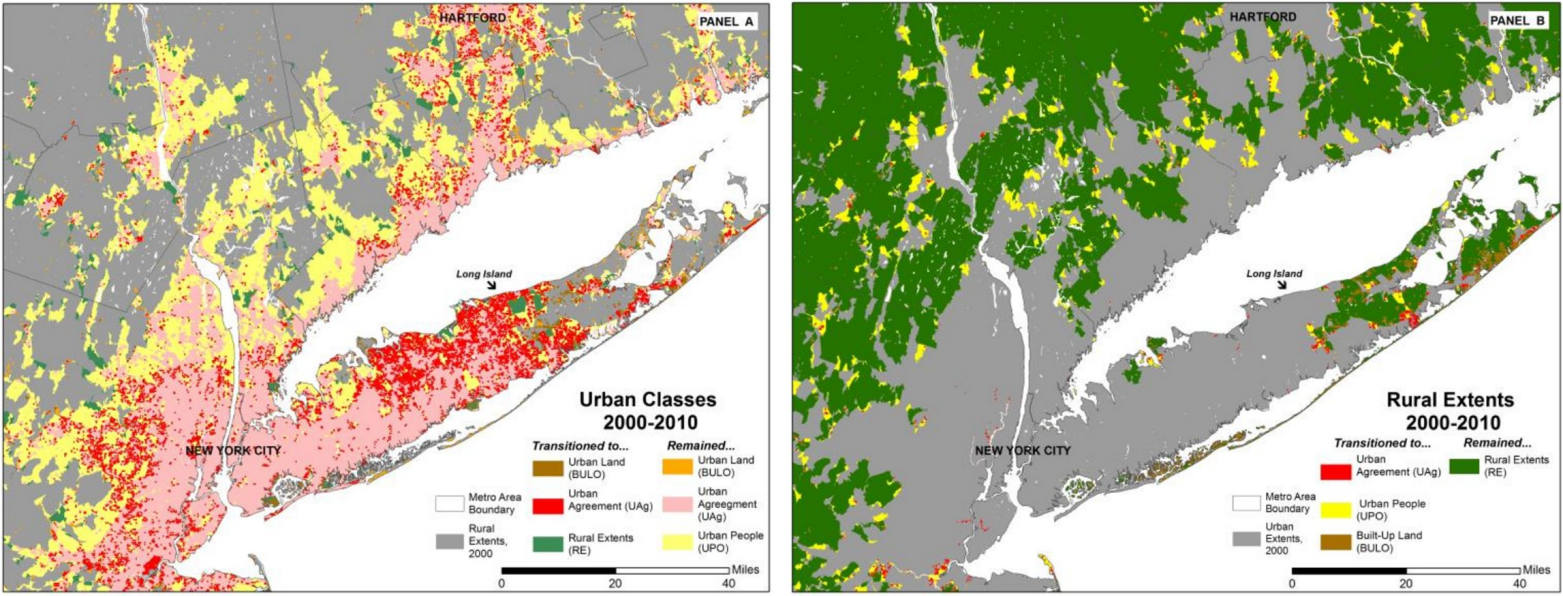
**Change in classifications, New York city MSA, 2000–2010: changes from 2000 all *urban* classes in panel A and from 2000 *rural extents* in panel B.** NB: As indicated in Table 5, no *UAg* or *BULO* area transitions to *UPO* (Panel A), but some *RE* area transitions to *UPO* (Panel B).

Table 5 documents these transitions; it can be read in conjunction with the illustrations in Fig 2, which shows, as an example, the dynamics of change for much of the New York City metro area (officially, the Metropolitan Statistical Area, MSA). Examining the transitions of *UI* land, Table 5 shows that in both 1990–2000 and 2000–2010, areas in any given class at the start of a decade are likely to remain in that class at its end. This is especially true for areas of *UAg*: 96 percent of the land area in this status in 1990 is again so classified in 2000; over the 2000–2010 period, 99 percent remained *UAg*. The relatively little area exiting this class almost always enters the class of built-up but not officially urban (*BULO*) a decade later; this occurs mostly on the edges of an urban area, as in Long Island (see Panel A of Fig 2). Of land area that is classified as *BULO* in 1990, 39 percent transitioned to the *UAg* class, thus retaining its built-up status but being newly designated as officially urban by the census. As seen in Fig 2, these are areas that can be described as either in-fill within larger urban areas, or localities on the edge of the existing area of *UAg*. From 2000–2010, more than half of the *BULO* area underwent this transformation. As with *UAg* areas, no land area classified as *BULO* was reclassified as *RE* in subsequent decades. Overall, therefore, the dynamics exhibited by the *BULO* areas suggest that built-up but not-yet-officially-urban areas may have significant potential to gain an official urban designation over the course of a decade, thus serving as a leading indicator of urbanizing population.

**Table 5:** Decadal change in urban classifications, and comparison to rural extents, national-level, 1990–2000 and 2000–2010.

	Area (km^2^)		Land Classification and Area, in the following decade (% of original area)				
	by Class and Year						
Urban Class	1990	2000	Layer	2000		2010	
Uag	78,216	89,508	UAg	74,964	(95.84)	88,847	(99.26)
			BULO	3,252	(4.16)	661	(0.74)
			UPO	0	(0)	0	(0)
BULO	10,717	13,740	UAg	4,200	(39.19)	7,025	(51.13)
			BULO	6,516	(60.81)	6,715	(48.87)
			UPO	0	(0)	0	(0)
UPO	146,869	150,677	UAg	7,368	(5.02)	8,786	(5.83)
			BULO	933	(0.64)	244	(0.16)
			UPO	89,484	(60.93)	119,194	(79.11)
			RE	49,084	(33.42)	22,453	(14.9)
RE	7,574,487	7,556,373	UAg	2,976	(0.04)	4,230	(0.06)
			BULO	3,039	(0.04)	3,245	(0.04)
			UPO	61,183	(0.81)	51,149	(0.68)
			RE	7,507,289	(99.11)	7,497,750	(99.22)

Among the less densely built-up urban localities (*UPO*), a significant fraction of the land area loses its official urban status to become low-density rural (*RE*) over a decade (33 percent make this transition from 1990–2000 and 15 percent over 2000–2010), with transitions to other classes being much less common. Inspecting these cases, we find that the areas which were reclassified as rural were initially somewhat more distant (1.4 and 2 km, in 2000 and 2010, respectively) from the nearest area of *UAg* than were those that remained *UPO* (which were about 0.5 km away). Additionally, the bulk of the area reclassified as *RE* was also subject to census block boundary changes between census years, suggesting that blocks are being reorganized to more cleanly separate their urban and rural components. Despite the sizable reclassification to *RE*, the majority of *UPO* land remains *UPO* land in the subsequent decade.

Table 5 and Panel B of Fig 2 also allow us to compare these transitions with those of land initially classified as rural. While only small fractions of *RE* land transition to any urban class over the course of a decade, the total amount of land in these classes is decidedly non-trivial. Most *RE* land that is reclassified transitions to the *UPO* class–some 60,000 km^2^ and 50,000 km^2^ respectively, in 2000 and 2010; when combined with transitions of *UPO* land to RE, this results in only a modest net gain of *UPO* land between 1990 and 2000 (≈12,000 km^2^), and a larger gain between 2000 and 2010 (≈29,000 km^2^). A decomposition of these transitions suggests that two distinct processes are at work: It appears that most of the *UPO* to *RE* transitions result from boundary changes whereby larger urban blocks with geographically condensed urban populations are split into urban and rural components. Conversely, *RE* to *UPO* transitions are associated with population growth. Significantly less *RE* land makes a transition to areas of *UAg* or *BULO*. However, the land that does transition to *BULO*–almost 3,000 km^2^ in each decade–comprises between 20 to 30 percent of land in the *BULO* class, 13,740 km^2^ and 10,865 km^2^, respectively, in 2000 and 2010. Further research will be required to delineate the full transition history of each land parcel (e.g., from *RE* to *UPO* or *BULO* to *UAg*), but it is clear that the transition of rural areas into urban ones is an important feature of the full continuum of the urbanization process, and is likely to be of considerable importance in non-US settings. For example, in China Zhu [44] has termed this process *in situ* urbanization. Finally, as with the transformations within urban classes, there is a pattern evident in the transformation of *RE* areas to urban ones. Panel B of Fig 2 shows that they tend to be on the outlying areas of existing urban areas, although dispersed transformations to both *BULO* and *UPO* areas are also notable.

## Discussion

In this analysis, the incorporation of remote sensing-derived, built-up land layer–such as those derived from GHSL–has enabled us to pursue three objectives. First, by combining census-defined urban areas with GHSL and in this way integrating demographic with land-cover research perspectives, we aimed to learn in a more holistic way about urban structure. The Census Bureau has taken steps in this direction as well, by including the impervious surface land cover class in its urban definitions for the 2010 census. Our results suggest that this blended approach is well worth pursuing. Second, the study aimed to identify the potential of using GHSL-built-up measures as a spatially and temporally consistent proxy for urban classes, which could be applied to urban land or urban people in regions and countries in which no (or quite limited) census-related urban classes exist. Third, we were interested in understanding the potential to use GHSL for creating urban indicators and in spatial forecasting of urban land. Our results will be discussed below in the light of these three objectives.

### Combined data layers reveal patterns of urban structure and urbanization

This study has created a new way of combining satellite-derived settlement layers with census data to model different urban classes, including areas where urban people live in urban-designated land (areas of urban agreement, *UAg*), built-up land only (*BULO*; in which the population is considered to be rural by the census) and where officially-designated urban people live on non-urban land–that is, on land that is not very built-up (*UPO*). These distinct, thematically refined classes provide a unique picture of the distribution of urban people and urban land and give insight into the variety of urban patterns across the whole nation–allowing us to characterize urban expansion and in-filling as well as urban densification over three censuses.

An overwhelming share of the urban population of the US (nearly 70 percent) live in areas of urban agreement, consistently throughout the 20 year period we study. Of all people living in built-up areas, very small percentages–only 1.5 percent in 1990, declining to 0.3 percent in 2010 –are not classified as urban by the census. Our analysis reveals that different classes have very different built-up levels. Areas of *UAg* are overwhelmingly built-up, being between 5–10 times more built-up than areas of *UPO* (depending on the decade and GHSL threshold considered). All urban classes have far greater built-up percentages than rural extents, reflecting in some part an imbalanced classification accuracy of GHSL [39], as well as the fact that officially rural areas include uninhabited areas (such as national parks).

However, the full extent of urban living is not captured by built-up density alone: Using a 50 percent threshold, we find that around 30 percent of the officially urban population does not live in built-up areas. Several factors combine to produce this gap between the official urban population and the GHSL-based proxy for it. First, as we have shown, the degree to which the official urban population is captured by GHSL is sensitive to the choice of the built-up threshold. Second, the gap may be partly explained by the use of proximity and connectivity measures to urban cores in Census Bureau’s urban definitions, which are meant to include people who live an “urban lifestyle”. A third contributing factor is the expansive peri-urban and ex-urban development that is found commonly in the US, but which is much less prevalent in other countries [30]. Our analysis reveals that these uncaptured areas have built-up levels that are much lower than 50 percent. It is worth noting that in related studies, it has been found that GHSL (and Landscan SL, a similar product) outperform other measure of built-up land in detecting more peripheral urban development, while there is general agreement across products in more heavily built-up core urban areas [45]. This suggests that, by any measure, urban people tend to live well beyond the bounds of heavily built-up land (see S2 Text and S1 and S2 Tables for analysis at alternative GHSL thresholds). Finally, the gap may also have something to do with our assumption that population is uniformly distributed within census blocks. We discuss alternatives to the uniformity assumption below.

Despite this shortcoming, the combination of block-level census classifications with GHSL enables us to describe urban patterns and their change over time in ways that cannot be done with more conventional, binary geographies, such as those of the temporally-changing MSA or CBSA classifications of metropolitan areas for the US. The fine-grained GHSL data offer additional information in the form of built-up proportion (which has an intuitive meaning related to the intensity of development), and is thus relevant for the identification and differentiation of urban land within any administratively-identified urban area. Other studies [4,11] have also grappled with this issue, but so far, as we are aware, ours is the first attempt to combine census urban classifications with remote-sensing derived data for the purpose of evaluating the degree of correspondence between such disparate data sources. GHSL data in and of themselves, and our combined classification scheme, both hold promise for use as urban extent proxies over time in settings where administrative data that include urban definitions are missing or badly out-of-date, or as in the case of MSA-type classifications, are too complex and time-variant to easily interpret.

### Can GHSL be used to derive consistent urban proxies in data-poor regions?

As we have shown, GHSL has very good potential in the measurement of urban processes not only across space but also across time. Although US census definitions have changed over time, it has been demonstrated that GHSL reflects urban areas and urban populations, consistently, in the sense that the degree of agreement between census-based classifications and GHSL-derived footprints remains essentially constant. These are encouraging results for the use of GHSL in measuring urban extents and change in non-U.S settings, enabling the analyst to maintain consistency in definitional criteria across countries and over time. However, it must also be cautioned that GHSL does not reflect the fullness of more complex urban definitions, as we see in the US. Nevertheless, it seems to capture the majority of population (our urban agreement class) that would be considered urban in other settings as well. Lower built-up thresholds capture a greater share of the urban population in the US, pointing to even greater potential in using GHSL at varying thresholds of 50 and less percent built-up.

### Can GHSL-based urban indicators be used in forecasting?

We found that most land area classified as urban agreement (*UAg*) in one decade remains in that class in the subsequent decades. The other two classes (*BULO* and *UPO*) both undergo some transition to areas of urban agreement, which is especially probable for areas of *BULO*. Understanding these transitions in full is part of a larger research agenda, but it seems that *BULO* and *UPO* areas might be viewed as leading indicators of change, and might therefore aid planners and analysts in preparing for urban transitions. In the present analysis, we find that although the population of *BULO* areas is quite small, over time these areas are increasingly likely (40 percent from 1990–2000 and 50 percent from 2000–2010) to become classified by the census as being urban. Only a small portion of the *UPO* land area transitions to *UAg*, roughly 5 percent in each of the two decades. Importantly, on the rural side of the urban-rural continuum, there are sizable transitions in both directions between *UPO* land and rural extents (*RE*), such that on net, the fraction of total population in *UPO* areas grows modestly over time. We cannot comment here on land that might have transitioned multiple times, as our analysis did not track the evolution of specific land parcels across all three census years. However the spatial distribution of transitions illustrated in Fig 2 indicate, for example, that transitions from *RE* to *UPO* often occur in close proximity to transitions from either *UPO* or *BULO* to *UAg*, suggesting a possible stepwise transition from *RE* up the urban hierarchy in some cases. While often thought of as a very American form of urbanization, this process may be consistent with that observed in a non-spatial analysis of European cities whereby total urban land expands while population growth, and in some instances even population totals, decline [46,47]. These observations suggest much potential for using GHSL to be predictive of densification as well as changes in urban structure over the relatively near term.

### Limitations of the study

There are several small but notable limitations of this study. First, we assume a uniform distribution of population across the territory of a census block, even when GHSL identifies only part of that block as built-up. Uniformity is a simplifying but flawed assumption: the true proportion of population within the built-up portion of such a block is likely to be higher. In adopting a uniform assumption, we are likely to have underestimated the population found in areas of *UAg* and *BULO* and over-estimated that of *UPO*. However, in this study we prioritized scalability to the whole U.S. over over the use of more complex dasymetric models to redistribute population to different parts within a block, which tends to be difficult to validate [48,49].

A second and closely related challenge has to do with boundary changes: census block geometries change across census years. The methodology applied in this study treated boundary changes at the block level as processes that may be part and parcel of urbanization (such as, administrative reclassification) and thus assumed comparability of block parts over time. The impact of this assumption in comparison to other methodological approaches is not yet known. (Future research will examine the role of block-level changes on urbanization.)

Third, the accuracy of GHSL in rural areas is known to be lower than in urban settings [39]. Because the *BULO* and *RE* classes are comprised only of areas that are officially rural, our estimates of *BULO* land area and population, and our estimates of GHSL values for both *BULO* and *RE* classes, may well be too low. As a consequence, the detection of urban area that does not meet the built-up proportion criterion is open to question.

Fourth, satellite data are inherently limited in that they cannot reveal anything about the key socioeconomic features of population composition—the domains of age, gender, education, race/ethnicity, and poverty—whereas census data do bring these critical dimensions to light, allowing them to be mapped together with population density and size. The vertical dimension of settlement is also clearly important, but to date no global data product—satellite-derived or social-science data—measures building volume (see [50,51] for examples of volumetric urban change for particular cities).

### Future research directions

Future research will explore more complex rules for built-up thresholds, perhaps by emulating the Census Bureau criteria on proximity, connectivity and density by allowing lower density built-up areas to be defined as proxy-urban if the area is within a certain distance of higher density areas. Such efforts would benefit from a comparative perspective to test the implications of built-up density specifications against a range of urban definitions and development patterns [1,6], so as to further advance efforts toward a global classification schema [30]. These advanced measures combined with higher-resolution satellites such as Sentinel in future GHSL releases [35] will further improve capturing built-up areas in more suburban, peri-urban and rural locations and thus help to reduce the discrepancy between areas that are only designated as urban by the census (*UPO*) and satellite-based urban measures.

It is important to undertake comparative work. We are currently conducting analysis comparing the results shown here with those for India and Mexico. Yet, the presented general approach can be adopted widely wherever there is access to fine-grained census (or even survey) data in which urban classifications are indicated. Further, since country-specific definitions of urban population have meaning, insight from a broad range of countries combined with in-depth knowledge of local understanding of urban forms will help to determine the potential limits of using satellite proxies.

Other ancillary data–such as night-time lights [52,53], historic housing and property data [54] and high-resolution satellite data that can differentiate among types of settlements–will be integrated in future research to examine how they can be used to refine estimates of the socioeconomic or demographic characteristics of place.

Methodologically, we will also make use of the distinct advantage of GHSL and similar high-resolution data to spatially refine census enumeration units, thus overcoming some of the persistent limitations in demographic analysis that typically assumes areal units to be internally uniform [55,56] and compares units that are inconsistent between censuses. This will directly benefit the application of areal interpolation methods [57,58] to create spatially refined and temporally consistent target units within which estimates can be compared more reliably.

Finally, future research will more formally investigate multi-temporal processes within the urban system. While the best methods to do this remain to be determined [16,59], in light of the fact that there are no existing spatial forecasts of city-growth or urbanization in this urban era, this is a promising new direction for scientific inquiry.

## Conclusions

This study has revealed important new aspects of the structure and composition of urban settings, showing how structure and composition are reflected in census data alone and satellite data alone, but are more fully revealed in their combination. Breaking from convention, we replace dichotomous indicators of urban-rural with graduated classes of land and population. We have found strong agreement between the US census urban classifications and the GHSL measures of built-up area, with the associations being constant over time and across GHSL thresholds. Areas of urban agreement–which meet both the census urban classification and GHSL threshold–are overwhelmingly built-up in comparison with areas that meet the census urban definition only. These are encouraging findings. Nevertheless, we would caution against the uncritical use of a single built-up GHSL threshold, which has the potential to misclassify the urban population. For the US case, we have shown that the adoption of a 50 percent built-up threshold (and a simple proportional allocation rule), fails to identify some 30 percent of the official urban population. Lower built-up thresholds capture more of the official urban population, and therefore merit careful consideration, as do richer specifications of the built-up surface that take contiguity and proximity into account. Combined census and satellite data can be further analyzed for even more detailed and nuanced characterizations of urban form and systematic evaluation of urban development patterns.

GHSL also holds promise for predictions of future urbanization and urban spatial patterns. This is a welcomed and long overdue advance in data and methods in both research and policy circles that have been dominated by use of simple aspatial trend interpolations/extrapolations of population estimates [1]. In the US, non-urban but substantially built-up places show a non-trivial likelihood of being classified as census-urban a decade later, suggesting that areas built-up but still officially classified as rural may represent a leading edge of urban change. Such *in situ* urban transitions may be taking place in other countries as well. The combination of built-up density data with ancillary data on roads and similar measures of connectivity [60] would seem to have excellent potential for forecasting urban change. Our research points to the need for focused and critical evaluation, especially of fringe areas and areas in transition.

## Supporting information

S1 Text Additional methodological detail. (DOCX)

S2 Text Sensitivity tests using alternative GHSL thresholds: Estimates of population and land area. (DOCX)

S1 Fig Constructing urban layers for the New York City MSA, including (a) urban blocks, (b) all urban land (GHSL 50% threshold), (c) urban inclusive area (UI), (d) urban agreement (UAg), (e) urban people only (UPO), (f) built-up land only (BULO), and (g) the entire urban hierarchy (including RE, UAg, UPO and BULO). Green background indicates rural extents (RE) in all maps.
(TIF)

S2 Fig Workflow for allocating land and population across urban classification scheme. (TIF)

S3 Fig Urban classifications, 1990 (upper) and 2010 (lower), with year-2000 MSA boundary, for selected pairs of large and small MSAs. (TIF)

S1 Table Population and land area by urban classification, 25% GHSL threshold. (DOCX)

S2 Table Population and land area by urban classification, 40% GHSL threshold. (DOCX)
