# Supplementary material for: Understanding urbanization: A study of census and satellite-derived urban classes in the United States, 1990-2010
Source: PLoS One. 2018 Dec 26;13(12):e0208487. doi: 10.1371/journal.pone.0208487 (PMC6306171; doi:10.1371/journal.pone.0208487)
Supplement: S1 Text — (DOCX) [file pone.0208487.s001.docx]

**Supplementary Information**

**S1. Additional methodological detail**

Corresponding to Fig 1 in the main text, S1 Fig shows different classes and class combinations for the New York City Metropolitan Statistical Area (MSA), at a GHSL built-up threshold of 50% and census data for the year 2010. This map sequence illustrates the steps that were taken to produce our spatial layers that form the backbone of this analysis: (1) We define people-based urban extents by adopting the census-based definition (**S1a** Fig). (2) Using the GHSL 304m resolution built-up land layer, we produce corresponding land-based estimates of developed extents for each of the mentioned thresholds. **S1b** Fig illustrates the distribution of developed land at the 50% built-up threshold. (3) We take the union of the people-based urban extents and land-based developed extents (i.e., with GHSL values greater than the given threshold) to generate a third layer, *urban inclusive (UI)*, which includes any land area meeting at least one of the two definitions (**S1c** Fig). (4) Any land not meeting any of these definitions is defined as *“rural extents” (RE)* indicated as green in **S1** Fig. We further decompose the *urban inclusive* (*UI*) class by distinguishing from one another people- and land-based conceptualizations through additional spatial overlay operations. (5) In order to create a class of *urban agreement (UAg)*, we intersect the people-based urban extents (census blocks) and land-based (GHSL) developed extents to produce a new layer where both census and built-up definition have been met (Fig 1 and **S1d**). (6) To create a class which meets only the census (people)-based definition of urban, we eliminate (i.e., erase) the area of *urban agreement* class from the people-based urban extent (Fig 1 and **S1e** Fig); entitled *urban people only (UPO)*. (7) We apply the same procedure a second time erasing the areas of urban agreement from the GHSL-based developed extent, extracting a layer we entitle *built-up land only (BULO)* in Fig 1 and **S1f** Fig. (8) Finally, the *UAg*, *UPO*, *BULO*, and *RE* layers are integrated to produce a four-class distribution across the entire area (Fig 1 and **S1g** Fig).

The details of the workflow for processing block-level (vector-format) census data and built-up (raster-format) data from GHSL to classify land and population according to this schema is given in S2 Fig. S3 Fig shows these classes for two metropolitan areas (New York and Atlanta), in 1990 and 2010. All geoprocessing was carried out using ArcGIS 10.4.1 software in a Python environment, and the procedure is repeated for each census year.
