# Supplementary material for: Understanding urbanization: A study of census and satellite-derived urban classes in the United States, 1990-2010
Source: PLoS One. 2018 Dec 26;13(12):e0208487. doi: 10.1371/journal.pone.0208487 (PMC6306171; doi:10.1371/journal.pone.0208487)
Supplement: S2 Text — (DOCX) [file pone.0208487.s002.docx]

**Supplementary Information**

**S2. Sensitivity tests using alternative GHSL thresholds:** **Estimates of population and land area**

Tables S1 and S2 show population and land area in each urban class, and rural extents, for 1990-2010 at the 25% (Table S1) and 40% (Table S2) GHSL thresholds, to determine the sensitivity of our estimation to the specified threshold. In general, as the GHSL threshold is lowered the total area and population designated as urban inclusive (*UI*) rises while population density falls, a function of increasing urban land (GHSL) into areas of lower population density.

At lower GHSL thresholds, the percent of the urban population living in areas of urban agreement (*UAg*) increases, as does the percentage of population living in areas of built-up land only (*BULO*). The percent of the population living in *UPO* areas, those that are typically suburban or peri-urban in nature, is much smaller at the 25% threshold (17.4%, in 2010) than at the 50% threshold (31.1%, in 2010, Table 2). In this way, the *UPO* class is the most sensitive one to threshold changes, which is relevant because *UPO* areas are those identified by the census as urban by not by the GHSL proxy. Lower, more inclusive GHSL thresholds envelop more census blocks (and their population and land area), leading to both higher fractions of the population in the *UAg* and *BULO* classes, but naturally reducing the population estimates in *UPO* areas.

At each threshold, trends across the three census periods are similar, with increasing values in urban inclusive population and area over time. Consistent with the 50% GHSL threshold, within the urban population, the portion living in areas of *UAg* is largely stable over time, while “suburban” populations (*UPO*) generally comprise a larger portion of the urban inclusive population over time. Over time, the portion of urban land classified as *UAg* grows, while the portion defined as *UPO* and *BULO* decline. It is notable that even at the 25% GHSL threshold, only 4% of land area in 2010 would be classified as *UI*, up from 3.7% at the 50% threshold (Table 2).
