# Supplementary figures and images for: Understanding urbanization: A study of census and satellite-derived urban classes in the United States, 1990-2010
Source: PLoS One. 2018 Dec 26;13(12):e0208487. doi: 10.1371/journal.pone.0208487 (PMC6306171; doi:10.1371/journal.pone.0208487)

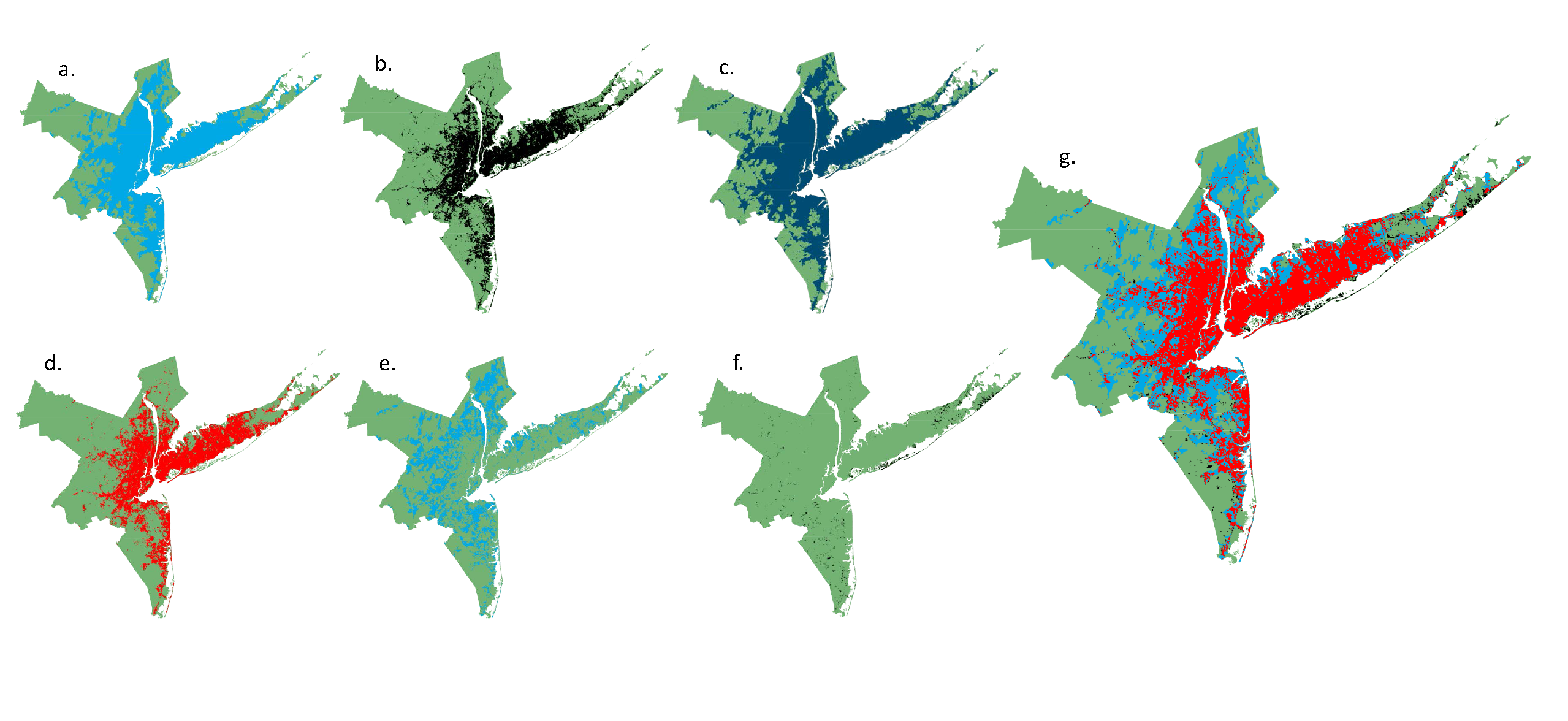

Supplement: S1 Fig — Constructing urban layers for the New York City MSA, including (a) urban blocks, (b) all urban land (GHSL 50% threshold), (c) urban inclusive area (UI), (d) urban agreement (UAg), (e) urban people only (UPO), (f) built-up land only (BULO), and (g) the entire urban hierarchy (including RE, UAg, UPO and BULO). Green background indicates rural extents (RE) in all maps. (TIF) [file pone.0208487.s003.tif]

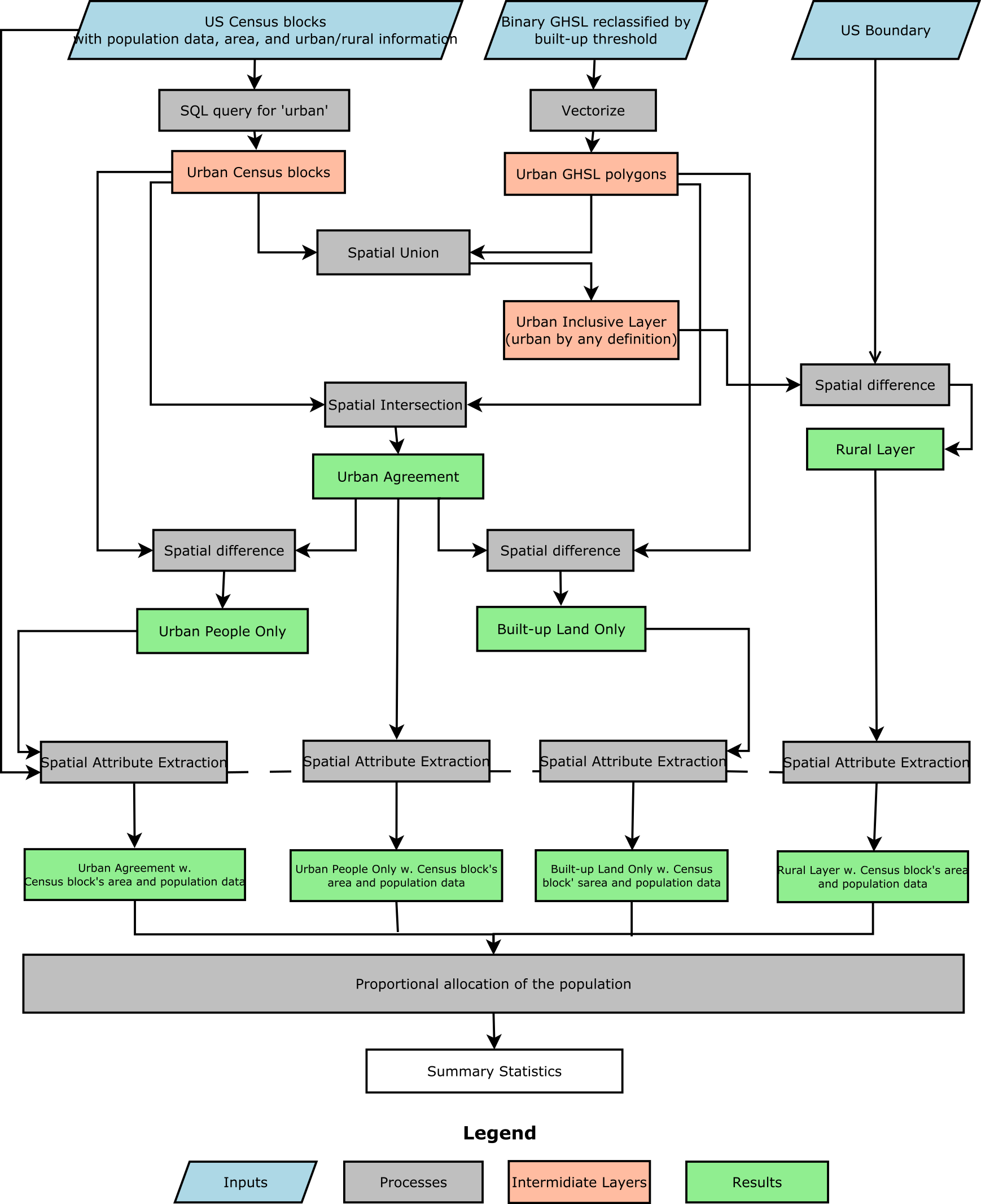

Supplement: S2 Fig — (TIF) [file pone.0208487.s004.tif]

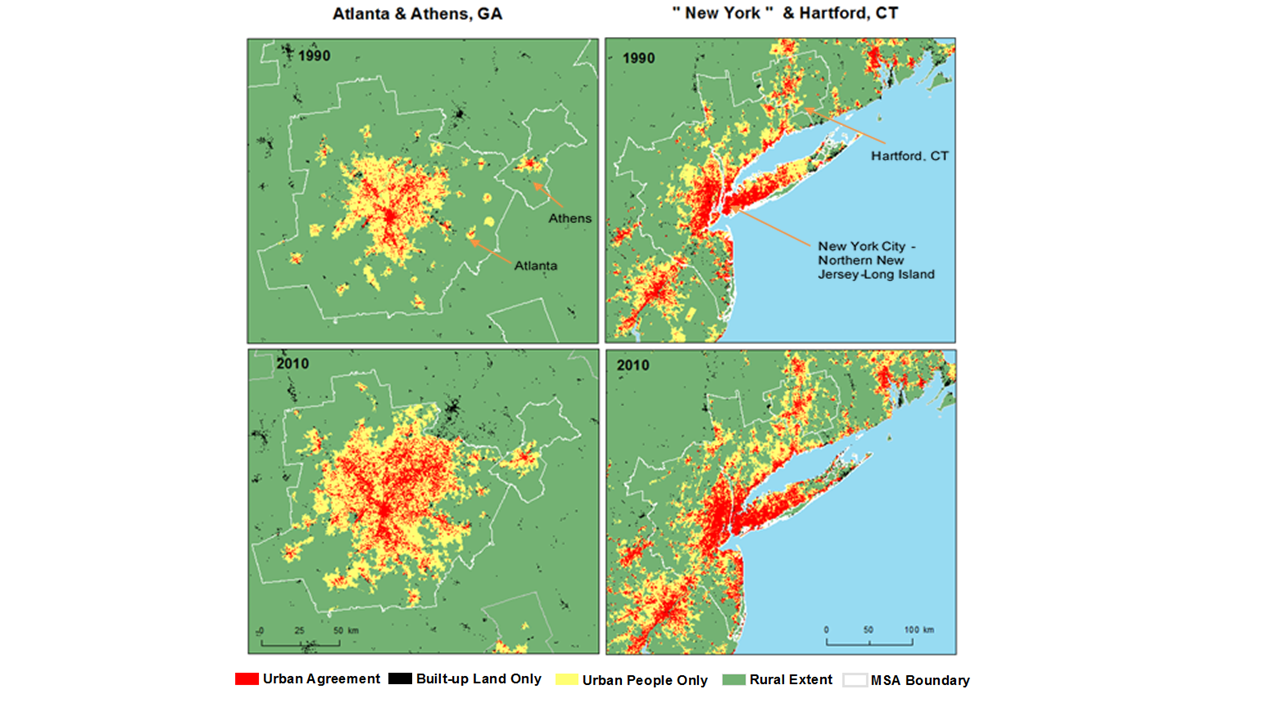

Supplement: S3 Fig — (TIF) [file pone.0208487.s005.tif]
