# Supplementary material for: Understanding urbanization: A study of census and satellite-derived urban classes in the United States, 1990-2010
Source: PLoS One. 2018 Dec 26;13(12):e0208487. doi: 10.1371/journal.pone.0208487 (PMC6306171; doi:10.1371/journal.pone.0208487)
Supplement: S1 Table — (DOCX) [file pone.0208487.s006.docx]

**Table S1. Population and land area by urban class, 25% GHSL threshold**

| **25% GHSL Threshold** | | **1990** | | **2000** | | **2010** | |
| --- | --- | --- | --- | --- | --- | --- | --- |
|  |  | Count | % | Count | % | Count | % |
| **Population (000s)** | **Urban Inclusive (*UI*)** | 192,318 | 77.9% | 225,289 | 80.6% | 251,870 | 82.1% |
|  | Urban Agreement | 154,953 | 80.6% | 181,381 | 80.5% | 203,793 | 80.9% |
|  | Urban People Only | 30,438 | 15.8% | 39,370 | 17.5% | 43,724 | 17.4% |
|  | Built-up land Only | 6,926 | 3.6% | 4,538 | 2.0% | 4,354 | 1.7% |
|  | **Rural Extents *(RE)*** | 54,419 | 22.1% | 54,294 | 19.4% | 54,805 | 17.9% |
| **Area (km²)** | **Urban Inclusive (*UI*)** | 259,058 | 3.3% | 280,432 | 3.6% | 316,232 | 4.0% |
|  | Urban Agreement | 112,384 | 43.4% | 130,023 | 46.4% | 158,554 | 50.1% |
|  | Urban People Only | 112,763 | 43.5% | 110,188 | 39.3% | 120,711 | 38.2% |
|  | Built-up land Only | 33,912 | 13.1% | 40,221 | 14.3% | 36,966 | 11.7% |
|  | **Rural Extents *(RE)*** | 7,551,230 | 96.7% | 7,529,866 | 96.4% | 7,494,067 | 96.0% |
| **Population Density (Persons/km²)** | **Urban Inclusive (*UI*)** | 742.4 |  | 803.4 |  | 796.5 |  |
|  | Urban Agreement | 1,378.8 |  | 1,395.0 |  | 1,285.3 |  |
|  | Urban People Only | 269.9 |  | 357.3 |  | 362.2 |  |
|  | Built-up land Only | 204.2 |  | 112.8 |  | 117.8 |  |
|  | **Rural Extents *(RE)*** | 7.2 |  | 7.2 |  | 7.3 |  |

**Table S2. Population and land area by urban class, 40% GHSL threshold.**

| **40% GHSL Threshold** | | **1990** | | **2000** | | **2010** | |
| --- | --- | --- | --- | --- | --- | --- | --- |
|  |  | Count | % | Count | % | Count | % |
| **Population (000s)** | **Urban Inclusive (*UI*)** | 189,505 | 76.8% | 223,320 | 79.9% | 249,898 | 81.5% |
|  | Urban Agreement | 140,994 | 74.4% | 164,103 | 73.5% | 183,955 | 73.6% |
|  | Urban People Only | 44,397 | 23.4% | 56,648 | 25.4% | 63,562 | 25.4% |
|  | Built-up land Only | 4,114 | 2.2% | 2,568 | 1.2% | 2,382 | 1.0% |
|  | **Rural Extents *(RE)*** | 57,232 | 23.2% | 56,264 | 20.1% | 56,777 | 18.5% |
| **Area (km²)** | **Urban Inclusive (*UI*)** | 241,680 | 3.1% | 260,715 | 3.3% | 295,919 | 3.8% |
|  | Urban Agreement | 90,609 | 37.5% | 104,162 | 40.0% | 127,050 | 42.9% |
|  | Urban People Only | 134,537 | 55.7% | 136,049 | 52.2% | 152,216 | 51.4% |
|  | Built-up land Only | 16,534 | 6.8% | 20,504 | 7.9% | 16,653 | 5.6% |
|  | **Rural Extents *(RE)*** | 7,568,608 | 96.9% | 7,549,583 | 96.7% | 7,514,380 | 96.2% |
| **Population Density (Persons/km²)** | **Urban Inclusive (*UI*)** | 784.1 |  | 856.6 |  | 844.5 |  |
|  | Urban Agreement | 1,556.1 |  | 1,575.5 |  | 1,447.9 |  |
|  | Urban People Only | 330.0 |  | 416.4 |  | 417.6 |  |
|  | Built-up land Only | 248.8 |  | 125.3 |  | 143.0 |  |
|  | **Rural Extents *(RE)*** | **7.6** |  | **7.5** |  | **7.6** |  |
