# Supplementary material for: Understanding urbanization: A study of census and satellite-derived urban classes in the United States, 1990-2010
Source: PLoS One. 2018 Dec 26;13(12):e0208487. doi: 10.1371/journal.pone.0208487 (PMC6306171; doi:10.1371/journal.pone.0208487)
Supplement: S2 Table — (DOCX) [file pone.0208487.s007.docx]

**Table S2. Population and land area by urban class, 40% GHSL threshold.**

| **40% GHSL Threshold** | | | **1990** | | | | **2000** | | | | **2010** | | | |
| --- | --- | --- | --- | --- | --- | --- | --- | --- | --- | --- | --- | --- | --- | --- |
|  |  |  | Count | | % | | Count | | % | | Count | | % | |
| **Population (000s)** | **Urban Inclusive (*UI*)** | 189,505 | | 76.8% | | 223,320 | | 79.9% | | 249,898 | | 81.5% | |  |
|  | Urban Agreement | 140,994 | | 74.4% | | 164,103 | | 73.5% | | 183,955 | | 73.6% | |  |
|  | Urban People Only | 44,397 | | 23.4% | | 56,648 | | 25.4% | | 63,562 | | 25.4% | |  |
|  | Built-up land Only | 4,114 | | 2.2% | | 2,568 | | 1.2% | | 2,382 | | 1.0% | |  |
|  | **Rural Extents *(RE)*** | 57,232 | | 23.2% | | 56,264 | | 20.1% | | 56,777 | | 18.5% | |  |
| **Area (km²)** | **Urban Inclusive (*UI*)** | 241,680 | | 3.1% | | 260,715 | | 3.3% | | 295,919 | | 3.8% | |  |
|  | Urban Agreement | 90,609 | | 37.5% | | 104,162 | | 40.0% | | 127,050 | | 42.9% | |  |
|  | Urban People Only | 134,537 | | 55.7% | | 136,049 | | 52.2% | | 152,216 | | 51.4% | |  |
|  | Built-up land Only | 16,534 | | 6.8% | | 20,504 | | 7.9% | | 16,653 | | 5.6% | |  |
|  | **Rural Extents *(RE)*** | 7,568,608 | | 96.9% | | 7,549,583 | | 96.7% | | 7,514,380 | | 96.2% | |  |
| **Population Density (Persons/km²)** | **Urban Inclusive (*UI*)** | 784.1 | |  | | 856.6 | |  | | 844.5 | |  | |  |
|  | Urban Agreement | 1,556.1 | |  | | 1,575.5 | |  | | 1,447.9 | |  | |  |
|  | Urban People Only | 330.0 | |  | | 416.4 | |  | | 417.6 | |  | |  |
|  | Built-up land Only | 248.8 | |  | | 125.3 | |  | | 143.0 | |  | |  |
|  | **Rural Extents *(RE)*** | **7.6** | |  | | **7.5** | |  | | **7.6** | |  | |  |
